# Infection Prevention Measures for Surgical Procedures during a Middle East Respiratory Syndrome Outbreak in a Tertiary Care Hospital in South Korea

**DOI:** 10.1038/s41598-019-57216-x

**Published:** 2020-01-15

**Authors:** Jiyeon Park, Seung Yeon Yoo, Jae-Hoon Ko, Sangmin M. Lee, Yoon Joo Chung, Jong-Hwan Lee, Kyong Ran Peck, Jeong Jin Min

**Affiliations:** 1 Department of Anesthesiology and Pain Medicine, Samsung Medical Center, Sungkyunkwan University School of Medicine, Seoul, Korea; 2 Division of Infectious Diseases, Department of Medicine, Samsung Medical Center, Sungkyunkwan University School of Medicine, Seoul, Korea

**Keywords:** Preventive medicine, Viral infection

## Abstract

In 2015, we experienced the largest in-hospital Middle East respiratory syndrome (MERS) outbreak outside the Arabian Peninsula. We share the infection prevention measures for surgical procedures during the unexpected outbreak at our hospital. We reviewed all forms of related documents and collected information through interviews with healthcare workers of our hospital. After the onset of outbreak, a multidisciplinary team devised institutional MERS-control guidelines. Two standard operating rooms were converted to temporary negative-pressure rooms by physically decreasing the inflow air volume (−4.7 Pa in the main room and −1.2 Pa in the anteroom). Healthcare workers were equipped with standard or enhanced personal protective equipment according to the MERS-related patient’s profile and symptoms. Six MERS-related patients underwent emergency surgery, including four MERS-exposed and two MERS-confirmed patients. Negative conversion of MERS-CoV polymerase chain reaction tests was noticed for MERS-confirmed patients before surgery. MERS-exposed patients were also tested twice preoperatively, all of which were negative. All operative procedures in MERS-related patients were performed without specific adverse events or perioperative MERS transmission. Our experience with setting up a temporary negative-pressure operation room and our conservative approach for managing MERS-related patients can be referred in cases of future unexpected MERS outbreaks in non-endemic countries.

## Introduction

The Middle East respiratory syndrome (MERS) is a serious acute respiratory disease caused by the MERS coronavirus (MERS-CoV), and the mortality rates in infected patients are estimated at 20–40%^1^. Since the first case report in Saudi Arabia in 2012^2^, MERS outbreaks have occurred mainly in Middle Eastern countries and a small number of imported cases arose in Europe, Asia, United States, and Africa^3–7^. From May to July 2015, South Korea experienced the largest MERS outbreak outside the Arabian Peninsula^8,9^. The South Korean outbreak resulted in 186 laboratory-confirmed MERS cases, 92 of which were associated with our tertiary care hospital^10,11^.

According to our institutional policy during the MERS outbreak, all elective surgeries for MERS-related patients were postponed when possible. However, several MERS-related patients inevitably required emergency operations under anesthesia. Two of the 92-MERS confirmed cases and four MERS-exposed patients underwent surgery. Although there is some literature regarding infection prevention during operative procedures for severe acute respiratory syndrome (SARS) coronavirus^12,13^, guidelines or references for MERS prevention during perioperative patient care were very limited. Therefore, we developed institutional guidelines for perioperative MERS infection prevention and we set up a temporary negative-pressure operating room.

In this globalized era, along with small and large outbreaks that persist in the Arabian Peninsula, MERS outbreaks may recur in any other regions, especially if a super spreader introduces a MERS infection to a high-volume healthcare facility, which is how the previous South Korea outbreak occurred^11^. Moreover, there may be very few hospitals that are prepared to provide perioperative care for MERS patients. Therefore, herein, we share our experience of providing infection prevention and control measures for surgeries for MERS-related patients in our hospital.

## Results

**Figure 1:**
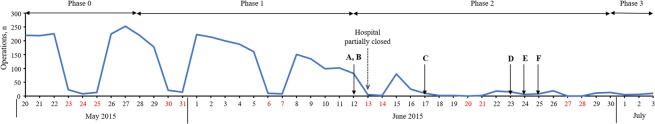
Total number of surgeries performed during the outbreak period at our hospital and the distribution of six MERS-related patients undergoing surgery.

During the MERS outbreak in our hospital, six MERS-related patients underwent surgery including three possibly exposed patients, one directly exposed patient, and two MERS-confirmed patients who recovered from the disease. All patients were negative during two preoperative MERS screenings using real-time reverse transcription polymerase chain reaction (rRT-PCR)^14^. Figure 1 shows the total number of surgeries performed during the outbreak period at our hospital and the distribution of MERS-related patients undergoing surgery.

### Temporary set-up of a negative-pressure operating room

The operating rooms in our hospital were generally positive-pressure environments, and we had no permanent negative-pressure operating rooms. Because a negative-pressure operating room is the optimal environment to prevent airborne virus spreading to adjacent areas^13^, two of our 25 operating rooms in the main operating suite of the hospital were temporarily converted into negative-pressure operating rooms to perform surgical procedures on MERS-related patients. Operating rooms no. 16 and 17 were selected because they were connected to each other, but each room had separate atmospheric air inlets and exhaust systems. They also had separate air-conditioning and humidification systems. Of the two connected rooms, one was used as the main operating room and the other was used as the anteroom where healthcare workers (HCWs) applied and removed the personal protective equipment (PPE).

**Figure 2:**
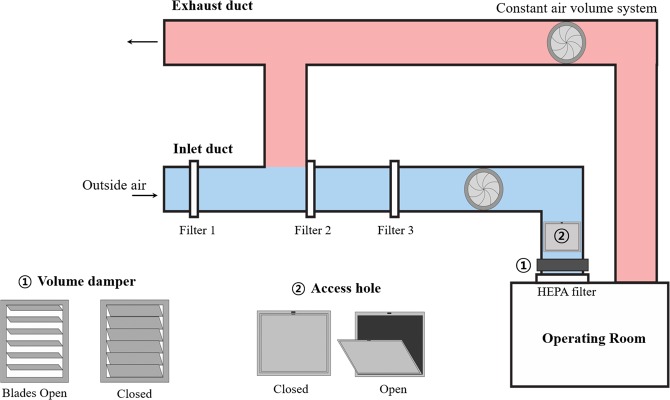
The ventilation system of the operation room.

**Figure 3:**
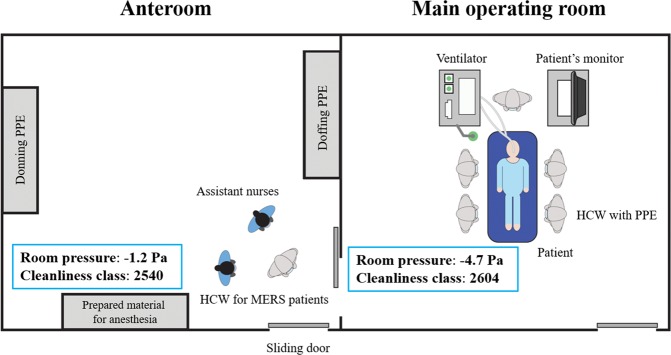
The temporary negative pressure operating room and the anteroom. Cleanliness class: The number of particles less than 0.5 um in 0.3048 m^3^; HCW: health care worker; PPE: personal protective equipment.

In each room, fresh air was supplied from an inlet duct and discharged outside through the exhaust duct (Fig. 2). Because a constant exhausting air volume was maintained through the outlet duct, negative pressure in the operating room was achieved by decreasing the inflow air volume that entered through the inlet duct. First, the blades of the air volume control damper in the inlet duct were closed as much as possible (Fig. 2). However, because the damper was not intended to be air-tight, the inflow volume to the operating room did not decrease sufficiently. Second, as an additional measure to decrease the inflow volume, we opened the access hole in the inlet duct, which was originally used for duct inspection purposes (Fig. 2). Finally, a smoke test was carried out to ensure negative pressure. The room pressure was maintained at −4.7 Pa in the main operating room and at −1.2 Pa in the anteroom (Fig. 3); −4.7 Pa is below the negative pressure room standard of −2.5 Pa^15^.

Airflow in both rooms reached 14–18 air exchanges per hour, according to airflow velocity measurements with an anemometer (EBT731 Balometer; TSI Alnor^®^, Minnesota, United States). In this environment, removing airborne contaminants requires 18 minutes for 99% efficiency and 28 minutes for 99.9% efficiency^16^. Therefore, 30 minutes of room ventilation was required after aerosol forming high-risk procedures, such as endotracheal intubation or extubation^12,17^. The cleanliness level of each room was also measured using a particle counter (TSI 9310; TSI, United States): main operating room = 2,604 and the anteroom = 2,540, which were much lower than the institutional target level of <10,000 for general surgery (Fig. 3). Cleanliness level was defined as the number of particles smaller than 0.5 µm in 0.3048 m^3^.

### Equipment preparation and disinfection

All built-in instruments such as computers, telephones, and ventilators were covered with plastic paper. Sufficient amounts of drugs, fluids, and other equipment were prepared in the operating room before surgery, and other unnecessary equipment was moved out. Additionally, we used disposable equipment, when possible. High efficiency particulate air (HEPA) filters were installed in the breathing circuits, both on the inspiratory and expiratory limbs of the ventilators and at the patient’s site that connected to endotracheal tube. After operations with MERS-exposed patients, 30 minutes of room ventilation was followed by surface disinfection with diluted chlorine bleach (500 ppm)^18,19^. Cleaners wore standard PPE while disinfecting surfaces. For MERS-confirmed patients, surface disinfection was performed twice.

### Institutional guidelines for perioperative management of MERS-related patients

During the MERS outbreak, we set the following principles for perioperative management of MERS-related patients: All elective surgeries for MERS-confirmed patients were postponed to reduce the risk of potential in-hospital transmission. For MERS-exposed patients, surgical procedures were delayed until after the potential incubation period of 14 days^20^. When a MERS-related patient required an urgent or emergency operation, MERS-CoV PCR tests were performed twice with distinct specimens preoperatively, to account for asymptomatic MERS patients or delayed positive conversion in symptomatic MERS-exposed patients. For patients with ambiguous PCR results or without a PCR test, operations were performed according to the management guidelines for MERS-confirmed patients. All the surgical procedures for MERS-related patients were performed in the last order of the day as possible.

### Perioperative protection level for HCWs

When an operation for a MERS-related patient was scheduled, the Division of Infectious Diseases and Infection Control Department confirmed the protection level of the HCWs, according to institutional guidelines (Table 1

**Table 1:** Composition of PPEs for perioperative protection of HCWs, according to the type of MERS-related patients and their symptoms.

MERS-related patients	MERS-related symptoms	PPE composition
**MERS-exposed**, possibly or directly	None	Standard* surgical gloves, surgical gown, eye shield, N95 respirator
	Present	**Enhanced** two pairs of surgical gloves (inner and outer), coverall clothes with head cover, shoe covers, goggles, PAPR or N95 respirator
**MERS-confirmed**	Asymptomatic or disappeared after recovery	
	Present	

*Enhanced PPE was applied for anesthesiologist regardless of type of patients or symptoms.

Abbreviations: PPE, personal protective equipment; HCW, healthcare worker; MERS, Middle East respiratory syndrome; PAPR, powered air purifying respirator.

). In principle, standard PPE was applied to HCWs who cared for asymptomatic MERS-exposed patients. Standard PPE includes surgical gloves, surgical gowns, eye shields, and N95 respirators. While managing MERS-confirmed or MERS-exposed patients with MERS-associated symptoms including fever, myalgia, respiratory symptoms, or diarrhea, HCWs implemented enhanced PPE, which included coverall clothes with head cover, shoe covers, goggles, two pairs of surgical gloves, and powered air purifying respirator (PAPR) or N95 respirators. Although we performed preoperative MERS-CoV PCR screening, enhanced PPE was still recommended when managing symptomatic MERS-exposed patients regardless of their PCR results. Anesthesiologists were recommended to apply enhanced PPE (including PAPR from the middle of the outbreak) when managing all MERS-related patients because they were most directly exposed to the aerosol-producing high-risk procedures, such as endotracheal intubation and extubation. Only minimal numbers of HCWs were present in the operating room. Institutional education regarding the precise use of PPE was provided to the all associated HCWs and they were assisted by skilled nurses in the operating room during the PPE donning and doffing processes.

### Patient transfer for operation

MERS-related patients were transferred directly to the negative-pressure main operating room through an exclusive path and elevator by a physician wearing proper PPE. The walls and the floor of the passageways and the elevator were covered with plastic paper. MERS-related patients wore a surgical mask during transfer. Because only anesthesiologists wore enhanced PPE when in proximity to asymptomatic MERS-exposed patients, 30 minutes of room ventilation was performed after anesthetic induction, including endotracheal intubation. The surgical team then entered the main operating room through the anteroom. In the cases of symptomatic MERS-exposed patients or MERS-confirmed cases, all HCWs wore enhanced PPE and the 30-minute ventilation time was not required.

**Figure 4:**
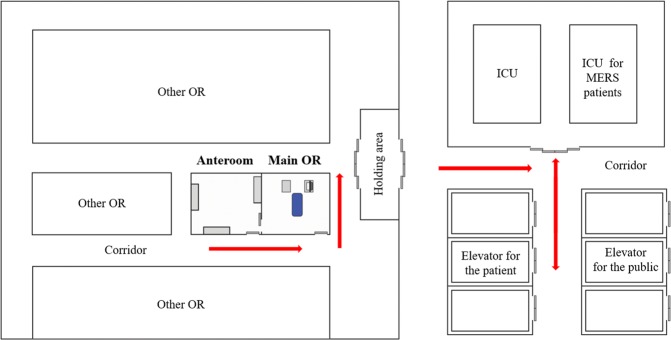
Transfer route for MERS-related patients. All pathways are closed during MERS-related patient transfer (red arrows). OR: operation room; ICU: intensive care unit; MERS: the middle east respiratory syndrome.

After completion of operation procedures, patients who were moved to the general ward recovered in the main operating room without going through the post-anesthesia care unit. Thirty minutes of room ventilation was performed after tracheal extubation. A physician in the main operating room sent the patient into the corridor, while the other physician outside the main operating room wearing PPE took over and transferred the patient to the general ward directly through the exclusive pathway (Fig. 4). Patients moving to the intensive care unit (ICU) were transferred while remaining intubated. Before transfers, we injected patients with a sufficient amount of intravenous muscle relaxant and sedative drugs to prevent coughing or movement and we applied a portable ventilator or bag-valve mask with a HEPA filter to the patient.

### Operations for six MERS-related patients

The details of the six MERS-related patients undergoing surgery are presented in Table 2. Two patients had operations during phase 1 and four patients during phase 2 of the outbreak (Fig. 1). The negative-pressure operating room was set up to be used from phase 2. Regarding PPE levels for the HCWs attending these six patients, standard PPE was applied during management of patient A (asymptomatic MERS-exposed patient), while anesthesiologists wore enhanced PPE for high-risk procedures (tracheal intubation). Enhanced PPE was applied to HCWs for patient B because the patient was symptomatic and still within the two-week incubation period, even though both PCR results were negative. Enhanced PPE, including PAPR to reduce risk of MERS-transmission, was applied for patient C who underwent surgery in the middle of the outbreak (phase 2). PAPR provides more perfect sealing and protection of the head surface.

**Table 2:** Detailed information for six MERS-related patients undergoing surgery during the MERS outbreak in our hospital.

Patient	Sex/age	Operation name/date	Type of MERS-relation	MERS-related symptoms	MERS-CoV PCR	PPE for HCWs	Negative pressure OR
A	F/67	Explore laparotomy/June 12	MERS-exposed (possibly)	None	Not done	Standard	No
B	M/12	Craniotomy and tumor removal/June 12	MERS-exposed (directly)	Fever	Negative twice	Enhanced	No
C	F/31	VA ECMO removal/June 12	MERS-exposed (directly)	Fever, persisting for 3 days	Negative twice	Enhanced	Yes
D	F/39	Caesarean section/June 23	MERS-confirmed and recovered	Myalgia, improving	Converted to negative, 3 days before surgery	Enhanced	Yes
E	M/16	Craniotomy and tumor removal/June 24	MERS-confirmed and recovered	None	Converted to negative, 12 days before surgery	Enhanced	Yes
F	F/59	Explore laparotomy/June 25	MERS-exposed (directly)	Fever, subsided	Negative once	Enhanced	Yes

Enhanced PPE included PAPR from patient C. Patient F underwent emergency operation of pan-peritonitis by colon cancer perforation, and the potential MERS incubation period of 14 days was passed at the time of operation.

Abbreviations: MERS, Middle East respiratory syndrome; CoV, coronavirus; PCR, polymerase chain reaction; PPE, personal protective equipment; HCW, healthcare worker; OR, operating room; VA, veno-arterial; ECMO, extracorporeal membrane oxygenation; PAPR, powered air purifying respirator.

Patients D and E had documented MERS-CoV infection and their recovery was confirmed with symptom resolution and two negative MERS-CoV PCR tests. However, enhanced PPE with PAPR was applied to HCWs because the infection risk could not be eliminated during exposure to a large amount of body fluid, especially during Cesarean section (patient D). After spinal anesthesia, she recovered in the main operating room and was transferred directly to the general ward. Patient F had a history of exposure to a MERS patient in the emergency room and was isolated due to a fever. Enhanced PPE was applied to the HCWs for patient F and she underwent surgery with only one set of negative PCR results because of her emergency condition.

All operative procedures for the MERS-related patients were performed without specific adverse events and there was no reported perioperative MERS transmission. The temporary negative-pressure operating room was maintained until June 25, 2015 and was converted back to two ordinary positive-pressure operating rooms after a declaration of the end of the MERS outbreak in our hospital.

## Discussion

MERS, as well as SARS, is associated with coronaviruses, both of which have high affinity for the lower repiratory tract and easily produce severe pneumonia^21–25^. Although MERS has lower human-to-human transmission potential and has resulted in fewer large outbreaks than SARS, there may be occasional amplification of clusters in healthcare settings^21–23^. Moreover, MERS case fatalities are reported to be much higher than SARS (35–45% for MERS and 10–15% for SARS)^12,17,23,24,26^. Unlike SARS, ongoing small and large MERS outbreaks in the Arabian Peninsula foster potential future MERS outbreaks in non-endemic countries. However, there is likely to be a very limited number of hospitals that are prepared with negative-pressure operating rooms, except for a few hospitals in Hong Kong that experienced the 2003 SARS outbreak^13^. Almost all hospitals generally have positive-pressure operating rooms and they may experience an outbreak without facilities that are prepared for perioperative management of MERS patients, as our hospital did in 2015.

One of the highlights of our experience during the outbreak was the temporary set-up of a negative-pressure operation room with an adequate pressure gradient (≥2.5 Pa) by modifying two connected operating rooms according to US Centers for Disease Control and Prevention (CDC) guidelines^15^. Continuous negative pressure was maintained in the main operating room (−4.7 Pa) and the anteroom (−1.2 Pa). This temporary setting was possible because the two adjacent rooms had separate atmospheric air inlets and exhaust systems. Although we could not measure the airflow pattern or dispersion of infectious particles directly^27^, the cleanliness levels in both operating rooms were 2,500 particles, well below the institutional target cleanliness for general surgery (<10,000 particles).

Although the precise route of MERS-virus transmission is currently not clearly understood^21^, MERS, as well as SARS, is known to spread by direct contact with infectious material, such as large respiratory droplets, and also by airborne routes^28,29^. Touching contaminated objects may also be a source of transmission; this is different from tuberculosis, which is transmitted by airborne routes^19,29^. Therefore, when performing procedures that generate aerosols, such as endotracheal intubation, in patients with MERS or SARS, HCWs must wear enhanced PPE, including gloves, a gown, either a face-shield that fully covers the front and sides of the face or goggles, and respiratory protection at least as protective as an N95 filtering face piece respirator^19,28,30^. When removing PPE, care should also be taken not to contact contaminated materials. Considering potential aerosol generation in operating rooms and the transmission risk of MERS-CoV while changing PPE, the temporary modification of an operating room to a negative-pressure room with an anteroom should provide suitable protection for HCWs participating in operations on MERS-related patients^5^.

A second highlight of our experience is the highly conservative application of PPE to HCWs. At the time of the outbreak, there were no specific guidelines for perioperative management^31^. Therefore, we used a conservative approach based on our experience and previous reports. First, although the previous guidelines recommended that asymptomatic MERS-exposed patients be managed as general patients undergoing surgery, we applied standard PPE to HCWs and we performed MERS-CoV PCR screening twice. Although MERS progressed gradually after symptom onset^32^, we could not exclude the possibility that asymptomatic MERS-exposed patients had the potential to develop symptomatic disease perioperatively. Moreover, we observed development of MERS after the known incubation period of 14 days in an immunocompromised host^10,33^; thus, the possibility of exceptional cases could be considered. Furthermore, a certain proportion of asymptomatic MERS-exposed patients could actually be asymptomatic MERS-infected patients. Approximately 21% of laboratory confirmed MERS patients have been classified as asymptomatic or having nonspecific mild symptoms at the time of testing^34^. The potential for transmission from asymptomatic MERS-CoV PCR-positive person is currently unknown, but there are reports about prolonged viral RNA detection in the upper respiratory tract in asymptomatic PCR-positive person^9^. Considering these points, it would be reasonable to prepare more conservatively than the existing guidelines call for.

Another point on which our preparations differed from the guidelines was the application of enhanced PPE, which emphasizes full protection of the body surface with a hooded coverall. During the outbreak in our hospital, MERS transmission events occurred among HCWs who were equipped with standard PPE, including N95 masks. Transmission may have occurred after possible contamination of uncovered head or face surfaces^35^. Therefore, if a patient with a MERS contact history had MERS-associated symptoms, applying enhanced PPE (either a N95 respirator or PAPR) during surgery would be appropriate for HCWs because numerous droplets and aerosols may be produced during airway interventions. Because the N95 may fit inadequately if worn for a long time or after movement during surgery, wearing PAPR will be more beneficial. However, unlike HCWs dealing with Ebola virus, impermeable and fluid-resistant gowns are not required because body fluids are not infectious as with Ebola virus diseases^9,28,35,36^.

Our experience was limited in that, as a MERS outbreak outside the endemic country, we did not have an opportunity to perform surgical procedures in actively virus-shedding MERS-infected patients. Additionally, our infection-prevention protocols would be too conservative to apply in MERS-endemic situations. However, considering the potential risk of infected HCWs, preventing MERS transmission is extremely important in the management of a MERS outbreak. Importantly, our experience can be generalized to other non-endemic countries for managing potential outbreaks of emerging respiratory diseases.

## Conclusion

In the era of globalization, a MERS outbreak can occur in any country outside the Middle East. A very limited number of hospitals are equipped with negative-pressure operating rooms, and therefore, most hospitals are likely to experience a MERS or other outbreak in an unprepared circumstance. We hope that this report will help other hospitals in preparing for future MERS outbreaks and infection control in unexpected conditions.

## Methods

This study was based on all available data at the Samsung Medical Center from the MERS outbreak and on interviews with HCWs associated with the outbreak. The study was approved by Samsung Medical Center Institutional Review Board. The documents for review included electronic medical records of MERS-related patients who underwent operative procedures and institutional guidelines for perioperative management of MERS-related surgical patients. The MERS guidelines were prepared through multidisciplinary team discussions that were held by our hospital’s infection control department during and after the MERS outbreak. The records about the temporary set-up of a negative-pressure operating room were also reviewed. We also collected data through interviews with HCWs who participated in surgery and anesthesia for MERS-related patients.

We defined MERS-related patients as those who were possibly or directly exposed to MERS or who had a previously confirmed MERS diagnosis^10^. In brief, patients who had a potential but unconfirmed close contact history with a MERS patient were defined as possibly exposed patients, and they were allowed to continue their normal activities until MERS-like symptoms developed. Directly exposed patients included those who had close contact with a known MERS patient and who did not wear proper PPE; these patients were isolated in their homes or in private negative-pressure rooms at our hospital.

Because the number of MERS-infected patients continuously increased at our hospital, we partially closed the hospital on June 13^10^, at which point outpatient-care clinics were closed and the emergency department was only available for life-threatening emergencies. All elective surgeries were postponed if possible^10^. We defined the early phase of the outbreak (before June 13) as phase 1 and the middle phase of the outbreak (from June 13) as phase 2.

For MERS-CoV PCR tests, either sputum or nasopharyngeal swab samples were collected and sputum samples were preferred if available^14^. Sputum was collected directly into a sterile, leak-proof, screw-capped sputum collection sterile container and nasopharyngeal swab was collected with an eSwab (482 C, Copan Diagnostics Inc., Murrieta, CA, USA). Clinical samples were screened by rRT-PCR testing with amplification targeting the upstream E region (upE) and confirmed by subsequent amplification of the open reading frame (ORF)1a using PowerChek™ MERS real-time PCR kits (Kogene Biotech, Seoul, Korea). All rRT-PCR reactions were performed using the 7500 Fast Real-Time PCR System (Applied Biosystems, Foster City, CA, USA). The PCR reaction was performed in a total volume of 20 μL (15 μL PCR reaction mixture and 5 μL template RNA). Thermocycling conditions included a step at 50 °C for 30 min, followed by 95 °C for 10 min and then 40 cycles of 15 s at 95 °C and 60 s at 60 °C. Positive viral template control and no-template control were included in each run. The glyceraldehyde-3-phosphate dehydrogenase (GAPDH) gene was amplified simultaneously as a heterologous internal control to monitor PCR inhibition. A positive test result was defined as a well-defined exponential fluorescence curve that crossed the cycle threshold (Ct) < 35 cycles for both upE and ORF1a. A sample was considered “equivocal” if the upE result was positive but the Ct value for ORF1a was >35 and <40. We interpreted the result as “indeterminate” if (1) the upE result was positive but the Ct value for ORF1a was undetected or if (2) the Ct value for upE was >35 and <40.

### Institutional review board statement

The study was performed in accordance with the declaration of Helsinki and experimental protocols were revised and approved by IRB at Samsung Medical Center. (IRB No. SMC 2016-08-156-002).

### Informed consent statement

IRB at Samsung Medical Center has approved the waiver of patient consent form because of the retrospective nature of this study. Patient confidentiality is maintained at all time in accordance with Samsung Medical Center policies.

## Data Availability

The datasets generated during and/or analysed during the current study are available from the corresponding author on reasonable request.
